# Supplementary material for: Structural Characterization of Mono and Dihydroxylated Umbelliferone Derivatives
Source: Molecules. 2020 Jul 31;25(15):3497. doi: 10.3390/molecules25153497 (PMC7436329; doi:10.3390/molecules25153497)
Supplement: Supplementary file 1 [file molecules-25-03497-s001.pdf]

Structural characterization of mono and dihydroxylated umbelliferone derivatives

Rubén Seoane-Rivero[1*], Estibaliz Ruiz-Bilbao[2], Rodrigo Navarro[3,4*], José Manuel Laza[5], José María Cuevas[1], Beñat Artetxe[2], Juan M. Gutiérrez-Zorrilla[2], José Luis Vilas-Vilela[5] and Ángel Marcos-Fernandez[3,4]

1 GAIKER Technology Centre, Basque Research and Technology Alliance (BRTA), Parque Tecnológico de Bizkaia, edificio 202, E-48170 Zamudio, Spain;
2 Departamento de Química Inorgánica, University of the Basque Country UPV/EHU, Apartado 644, 48080 Bilbao, Spain
3 Instituto de Ciencia y Tecnología de Polímeros (CSIC), Juan de la Cierva 3, 28006 Madrid, Spain.
4 Interdisciplinary Platform for "Sustainable Plastics towards a Circular Economy" (SUSPLAST-CSIC), Madrid, Spain
5 Departamento de Química Física, University of the Basque Country UPV/EHU, Apartado 644, 48080 Bilbao, Spain
* Correspondence: seoane@gaiker.es; rnavarro@ictp.csic.es

1. Materials

Deuterated chloroform ($CDCl_3$), deuterated dimethylsulfoxide (DMSO-d^6), resorcinol ($C_6H_6O_2$), ethyl acetoacetate ($C_6H_{10}O_3$, EtOAc), 2-bromoethanol (C_2H_5BrO), anhydrous pyridine (C_5H_5N), 4-dimethylaminopyridine (DMAP) and Dowex® H+ resin were supplied by Sigma–Aldrich, and used as received. Dichloromethane (CH_2Cl_2), 1,4-dioxane ($C_4H_8O_2$), concentrated sulphuric acid (H_2SO_4), ethyl acetate ($C_4H_8O_2$, EtOAc), ethanol (C_2H_5OH, EtOH), N,N-dimethylformamide (C_3H_7NO, DMF), potassium carbonate (K_2CO_3), sodium bisulphite ($NaHSO_3$), sodium carbonate (Na_2CO_3), brine solution, anhydrous magnesium sulphate ($MgSO_4$), toluene (C_7H_8), diethyl ether ($(C_2H_5)_2O$) and methanol (CH_3OH, MeOH) were supplied by Scharlau, and used as received.

2. Synthesis of coumarin derivatives

2.1 Synthesis of 7-hydroxy-4-methylcoumarin (HMC)

HMC was synthesized according to previous literature [1,2]. Resorcinol (11.0 g, 0.1 mol) and ethyl acetoacetate (13.0 g, 0.1 mol) were completely dissolved in 40 mL 1,4-dioxane. Then, concentrated sulphuric acid (3 mL) was dropwise added to the system, which was subsequently warmed up to 65ºC for 3 h as can be seen in Scheme S1. Afterwards, the suspension was cooled down to room temperature and poured into 300 mL ice water to obtain a yellowish precipitate. The crude product was dried in a vacuum oven and recrystallized twice in ethyl acetate to obtain white crystals of HMC with a yield of 70%, which was kept away from light. Colourless crystals suitable for single-crystal X ray diffraction analysis were obtained from ethyl acetate/ethanol (3:1) mixture. The 1H and 13C-NMR spectra are collected in Figure S1 and ATR-FTIR and its MS spectra are collected in Figure S2.

^1H NMR (300 MHz, DMSO-d^6, δ): 10.49 (s, 1H, –OH), 7.57 (d, J= 8.7 Hz, 1H, Ar–H), 6.78 (dd, J= 8.7 Hz J=2.3, 1H, Ar–H), 6.68 (d, J=2.3 Hz, 1H, Ar–H), 6.11 (s, 1H, C–H), 2.36 (s, 3H, $–CH_3$).

^{13}C NMR (101 MHz, DMSO-d^6, δ): 161.24, 160.44, 154.91, 153.56, 126.58, 112.94, 112.09, 110.34, 102.27, 39.52, 18.19.

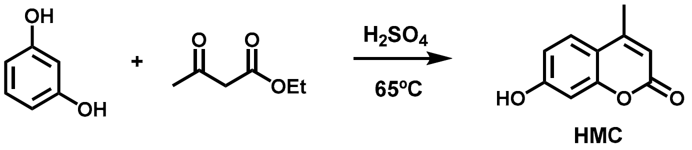

Scheme S1:Synthetic route for the preparation of HMC.

2.2 Synthesis of 7-(2-hydroxyethoxy)-4-methylcoumarin (HEOMC)

The synthetic procedure for HEOMC is described as follows (Scheme S2), according to previous literature [3]. In a 150 mL two-neck round-bottom flask, (4 g, 2.3×10^{-2} mol) of previously synthesized HMC was dissolved in 20 mL N,N-dimethylformamide. Then, a solution of (4.3 g, 3.4×10^{-2} mol) of 2-bromoethanol in 10 mL DMF was dropwise added to the reaction and (6.3 g, 45.6×10^{-2}mol) potassium carbonate was added one-off under stirring. The reaction mixture was stirred for 18 h at 88ºC under an argon atmosphere. The reaction was monitored by

TLC. When the reaction was completed, the mixture was cooled down to room temperature, poured into 70 mL ice water and filtrated to get the crude product. The solid was then recrystallized twice in ethyl acetate to obtain colourless crystals of HEOMC suitable for single crystal X-ray diffraction analysis with a yield of 86%. The 1H and 13C-NMR spectra are collected in Figure S3 and ATR-FTIR and its MS spectra are collected in Figure S4.

^{1}H NMR (400MHz, DMSO-d6, δ): 7.49 (d, 1H; J=8.8 Hz, Ar-H), 6.86 (dd, 1H; J_1=8.8 Hz, J_2=2.4 Hz, Ar-H), 6.81 (d, 1H, J=2.4 Hz, Ar-H), 6.13 (s, 1H, -CH), 4.53 (t, 2H, J=4.8 Hz, Ar-O-CH_2-), 4.25 (t, 2H, J=4.8 Hz, -CH_2-OCO-), 4.19 (d, 2H, J=11.2 Hz, -CH_2-OH), 3.64 (d, 2H, J=11.2 Hz, -CH_2-OH), 2.39 (s, 3H, -CH_3), 1.08 (s, 3H, -CH_3).

^{13}C NMR (101 MHz, DMSO-d6, δ): 161.79, 160.17, 154.69, 153.36, 126.34, 113.00, 112.39, 111.06, 101.12, 70.29, 59.39, 39.50, 18.12.

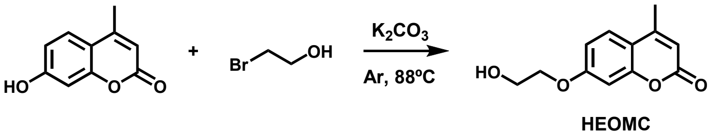

Scheme S2: Synthetic route for the preparation of HEOMC

The synthesis of both DHMC and DHEOMC require the synthesis of the same intermediate compound, isopropylidene-2.2-bis-(methoxy)propionic anhydride (DMPAA), which was synthesized according to literature [4,5].

2.3 Synthesis of 4-methylcoumarin-7-yl-3-hydroxy-2-(hidroxymethyl)-2-methylpropanoate DHMC

Then, DMPAA and HMC were reacted to produce an ester. The synthesis of the coumarin diol derivative was carried out following the route presented in Scheme S3. 7-hydroxy-4-methylcoumarin (HMC) (15.4 g, 8.6×10^{-2}mol) were added together with 4-dimethylaminopyridine (DMAP) (2.1 g, 1.7×10^{-2} mol) and dissolved in 238 mL of anhydrous pyridine and diluted with 537 mL of dichloromethane. The anhydride (DMPAA) (43.4 g, 13.1×10^{-2} mol) were added and reacted under stirring for 9 hours at room temperature. The excess of the anhydride was decomposed by stirring the reaction overnight (18 h) with 85 mL of a pyridine: water solution in a 1: 1 ratio. Subsequently, the following washes of the organic phase were carried out: 1M sodium bisulphite, sodium carbonate 10 % (w/t) and brine solution. The organic phase was dried over anhydrous magnesium sulphate and the solvent was removed at low pressure. 25.4 g (0.076 mol) of isopropyl-[2,2-bis-(methoxy)]-propanoate of 4-methylcoumarin were obtained, with a yield of 68%.

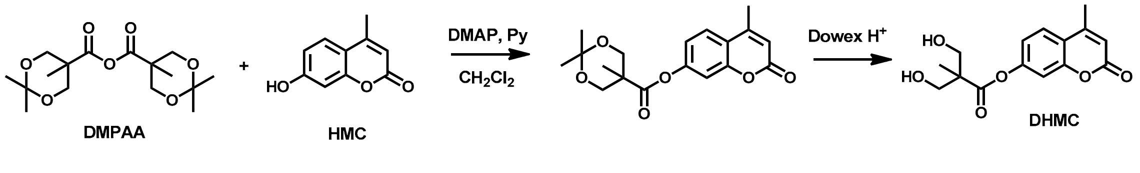

Scheme S3: Synthetic route for the preparation of DHMC.

Finally, the protecting group was easily removed yielding the HMC ester diol. The compound synthesized in the previous step (HMC-ester) was weighted, (25.4 g, 7.6×10^{-2}mol), and dissolved in 500 mL of methanol. Six teaspoon of the Dowex H^+ resin was added, and the mixture was stirred at room temperature for 3 hours. The resin was filtered and washed with 50 mL of methanol. The organic solvent was evaporated on a rotary evaporator and a white solid was obtained. The product was purified twice by recrystallization in ethyl acetate, obtaining a final yield of 30%. Then, the solid was recrystallized twice in ethyl acetate to obtain colourless crystals suitable for single crystal X-ray diffraction analysis. The 1H and 13C-NMR spectra are collected in Figure S5 and ATR-FTIR and its MS spectra are collected in Figure S6.

^{1}H NMR (400MHz, DMSO-d6, δ): 7.82 (d, J= 2.2Hz, 1H, Ar-H), 7.11 (m, 2H, Ar-H), 6.38 (s, 1H, -CH), 3.65 (m, 2H, OH), 3.51 (m, 4H, $-CH_2$-O), 2.44 (s, 3H, $-CH_3$), 1.17 (s, 3H, $-CH_3$).

^{13}C NMR (101 MHz, DMSO-d6, δ): 173.33, 159.60, 153.50, 153.35, 152.96, 126.33, 118.50, 117.37, 113.67, 110.00, 63.99, 51.23, 39.50, 18.18, 16.80.

2.4 Synthesis of 2-((4-methylcoumarin-7-yl)oxy)ethyl 3-hydroxy-2-(hydroxymethyl)-2-methylpropanoate DHEOMC

The synthesis of the DHEOMC followed the same procedure than the synthesis of the DHMC and it is shown in Scheme S4.

7-hydroxyethoxy-4-methylcoumarin (HMC) (15.1, 0.07 mol) were dissolved together with 4.16 g (0.03 mol) of 4-dimethylamino pyridine (DMAP) with a mixture of 138 mL of anhydrous pyridine and 554 mL of dichloromethane. 34 g (0.1 mol) of anhydride (DMPAA) were slowly added and left to react for 9 hours at room temperature. The excess of the anhydride was destroyed by stirring the reaction overnight with 66 mL of a pyridine:water solution in a 1:1 ratio. Subsequently, the organic phase was extracted consecutively with 1 M sodium bisulphite, 10%, sodium carbonate and brine solution. Finally, the organic phase was dried over anhydrous magnesium sulphate, which was then removed by filtration and the resulting solution was evaporated on a rotary evaporator, leaving a viscous orange solid. The yield was 73%.

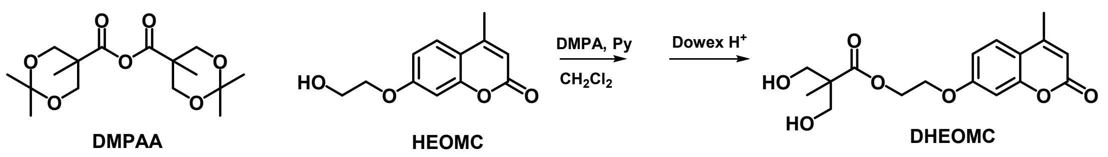

Scheme S4: Synthetic route for the preparation of DHEOMC.

For the deprotection step, 20.9 g (6.021 mmol) of the 4-methylcoumarin-7-ethoxyl ester of 2,2,5-trimethyl-[1,3] dioxan-5-(yl)acetic acid in 200 mL of methanol (MeOH) were dissolved and they added approximately 10 g of Dowex H^+ resin. The reaction was allowed to react at room temperature with stirring for 3 hours, the resin was filtered and washed with methanol. The methanol was evaporated on a rotary evaporator to give the desired compound (white crystals). The product obtained was purified by recrystallization from toluene. Colourless crystals suitable for single-crystal X ray diffraction analysis were obtained from ethyl acetate/diethyl ether (1:1) mixture. The 1H and 13C-NMR spectra are collected in Figure S7 and ATR-FTIR and its MS spectra are collected in Figure S8.

^1H RMN (400MHz, DMSO-d^6, δ): 7.51 (d, 1H; J=8.8 Hz, Ar-H), 6.87 (dd, 1H; J1=8.8 Hz, J2=2.4 Hz, Ar-H), 6.83 (d, 1H, J=2.4 Hz, Ar-H), 6.15 (s, 1H, CH), 4.56 (t, 2H, J=4.8 Hz, OCO-CH_2), 4.27 (t, 2H, J=4.8 Hz, CH_2-O-Ar), 3.92 (d, 2H, J=11.2 Hz, -CH_2OH), 3.74 (d, 2H, J=11.2 Hz, -CH_2OH), 2.40 (s, 3H, -CH3), 1.08 (s, 3H, -CH3).

^{13}C NMR (101 MHz, DMSO-d^6, δ): 174.70, 161.36, 160.13, 154.67, 153.35, 126.48, 113.31, 112.48, 111.29, 101.40, 66.64, 63.81, 62.04, 50.33, 39.50, 18.14, 16.84.

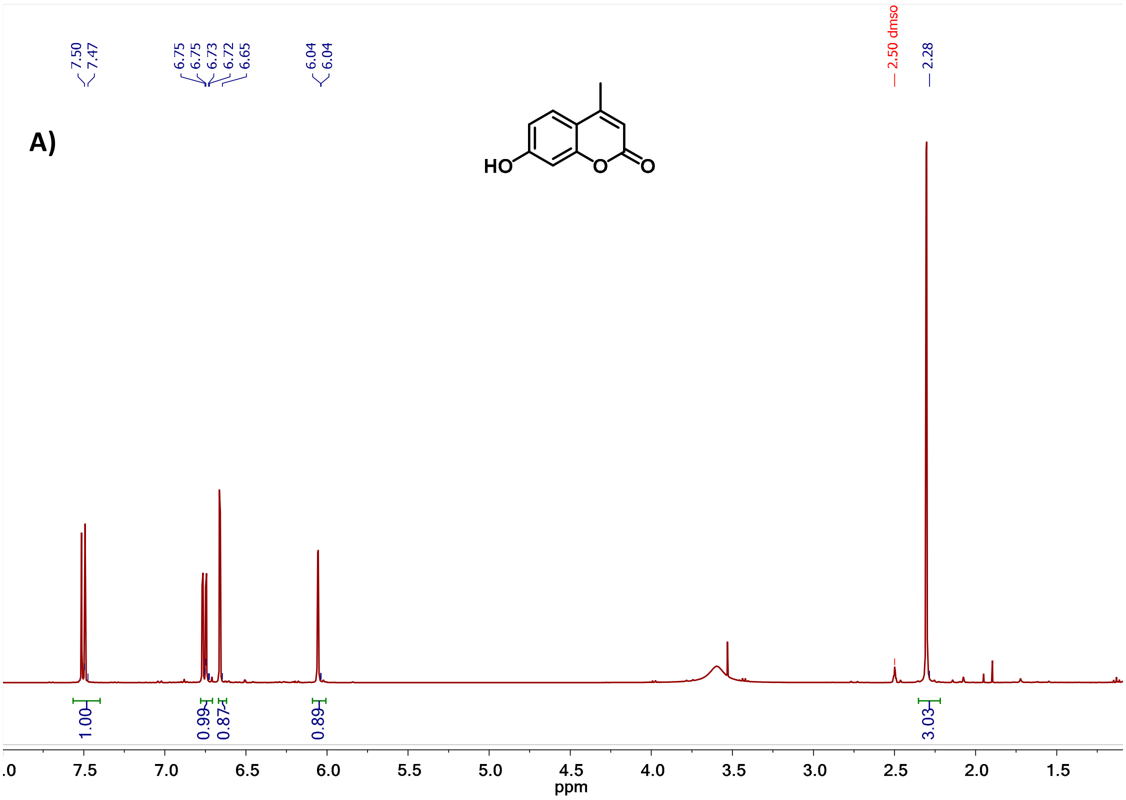

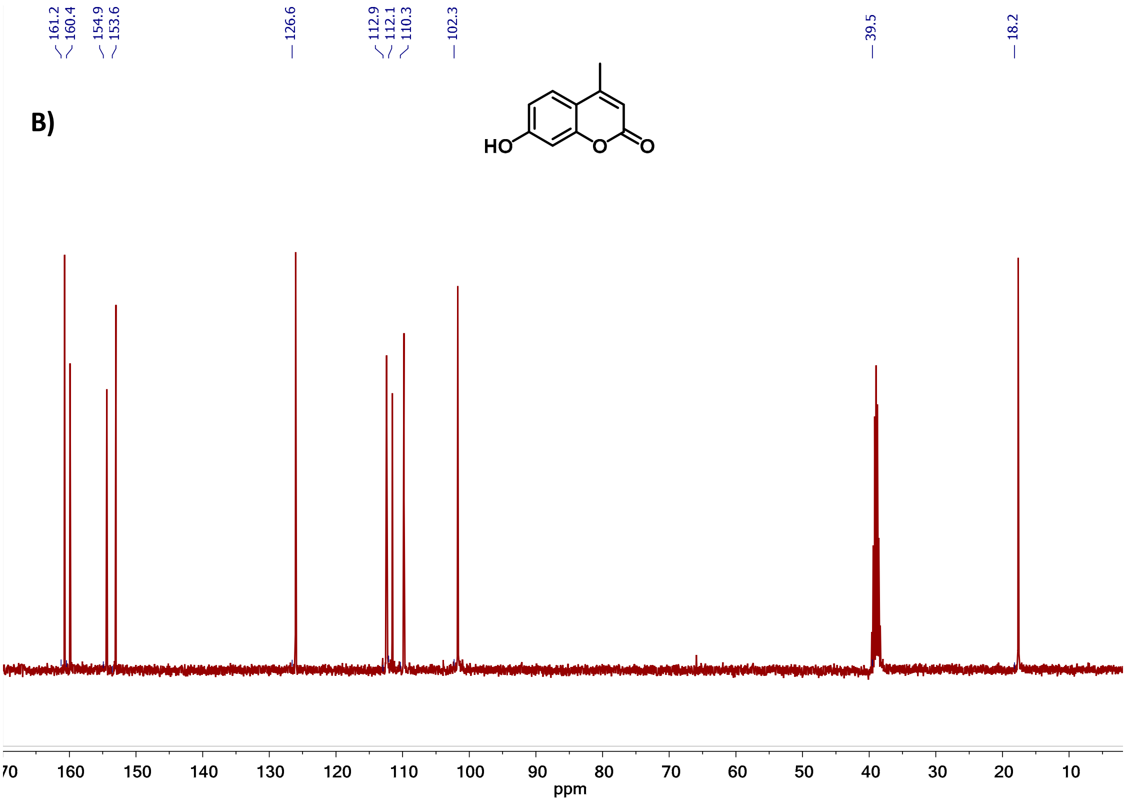

Figure S1: A) 1H-NMR and B) 13C-NMR spectra of HMC.

A)

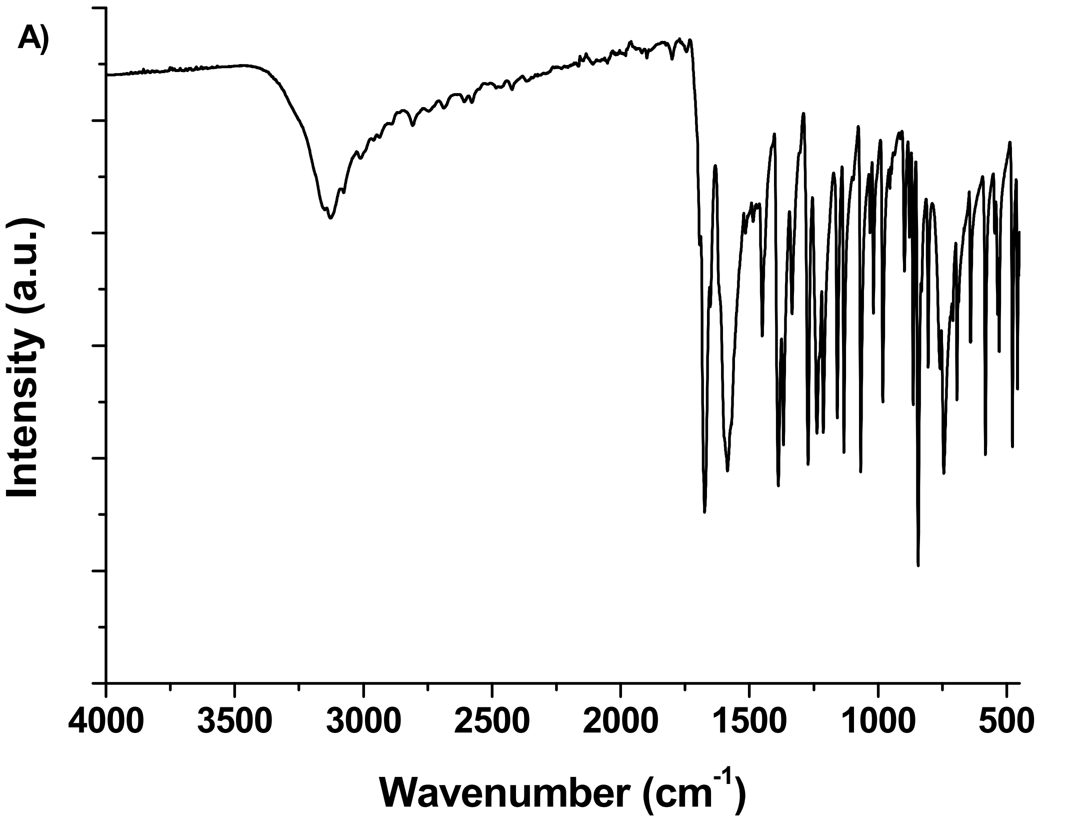

Compound Table

Compound Label	RT	Mass	Abund	Formula	Tgt Mass	Diff (ppm)
Cpd 1: C10 H8 O3	0.536	176.0473	27792	C10 H8 O3	176.04734	-0.26

B)

Compound Label	RT	Algorithm	Mass
Cpd 1: C10 H8 O3	0.536	Find By Formula	176.0473

MS Zoomed Spectrum

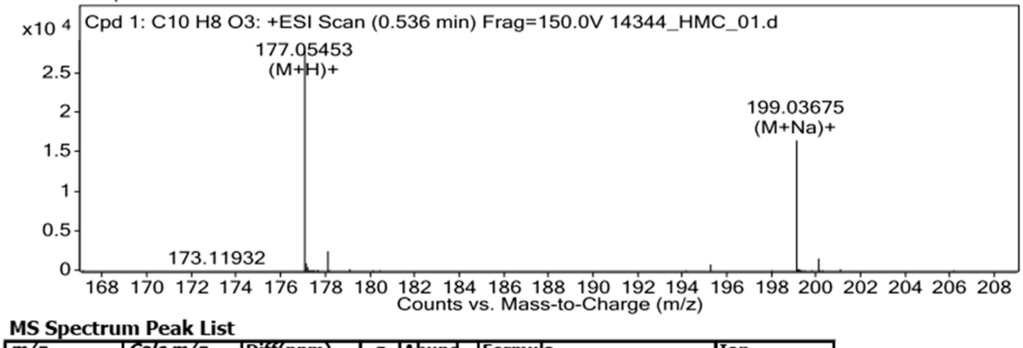

MS Spectrum Peak List

m/z	*Calc m/z*	Diff(ppm)	z	Abund	Formula	Ion
177.05453	177.05462	-0.53		27792	C10 H9 O3	(M+H)+
177.09319				1028		
177.14197				354		
177.17808				620		
177.27521				171		
178.05903	178.05801	5.72		2590	C10 H9 O3	(M+H)+
179.05683	179.06007	-18.09		299	C10 H9 O3	(M+H)+
199.03675	199.03657	0.94	1	16542	C10 H8 Na O3	(M+Na)+
200.03935	200.03995	-3.02	1	1724	C10 H8 Na O3	(M+Na)+
201.04329	201.04201	6.38	1	230	C10 H8 Na O3	(M+Na)+

Figure S2: A) ATR-FTIR and B) Mass spectra of HMC

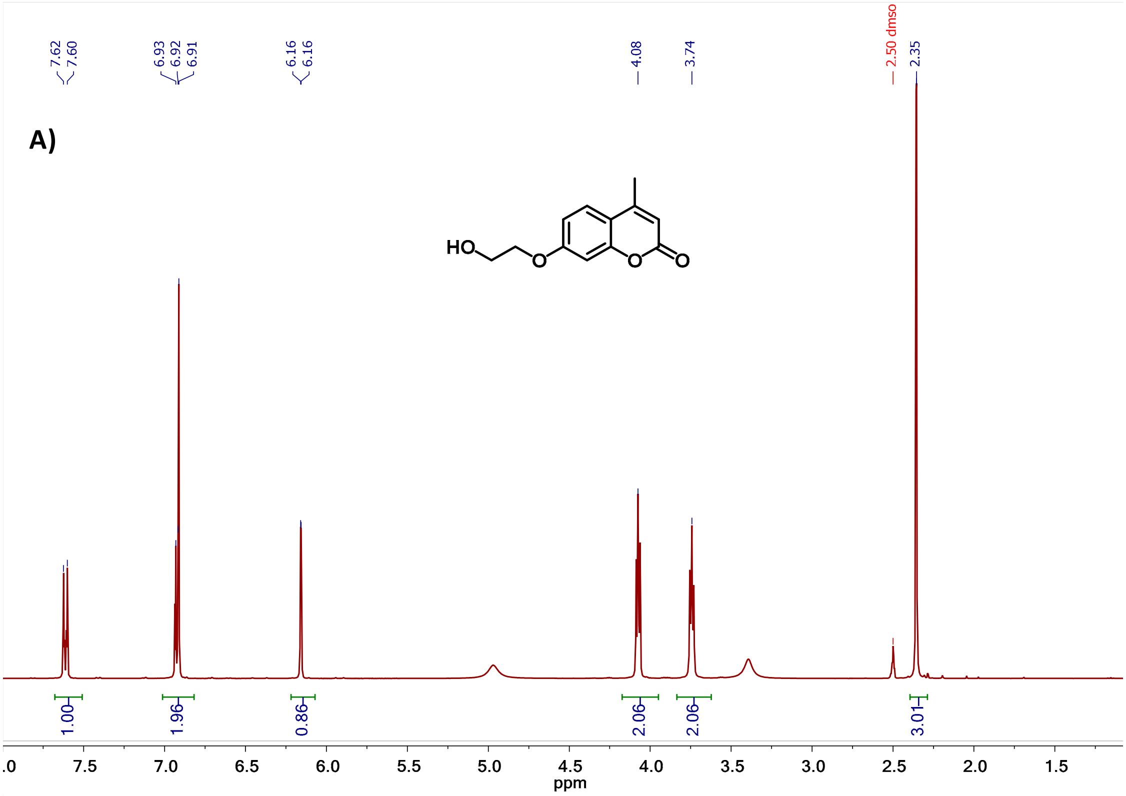

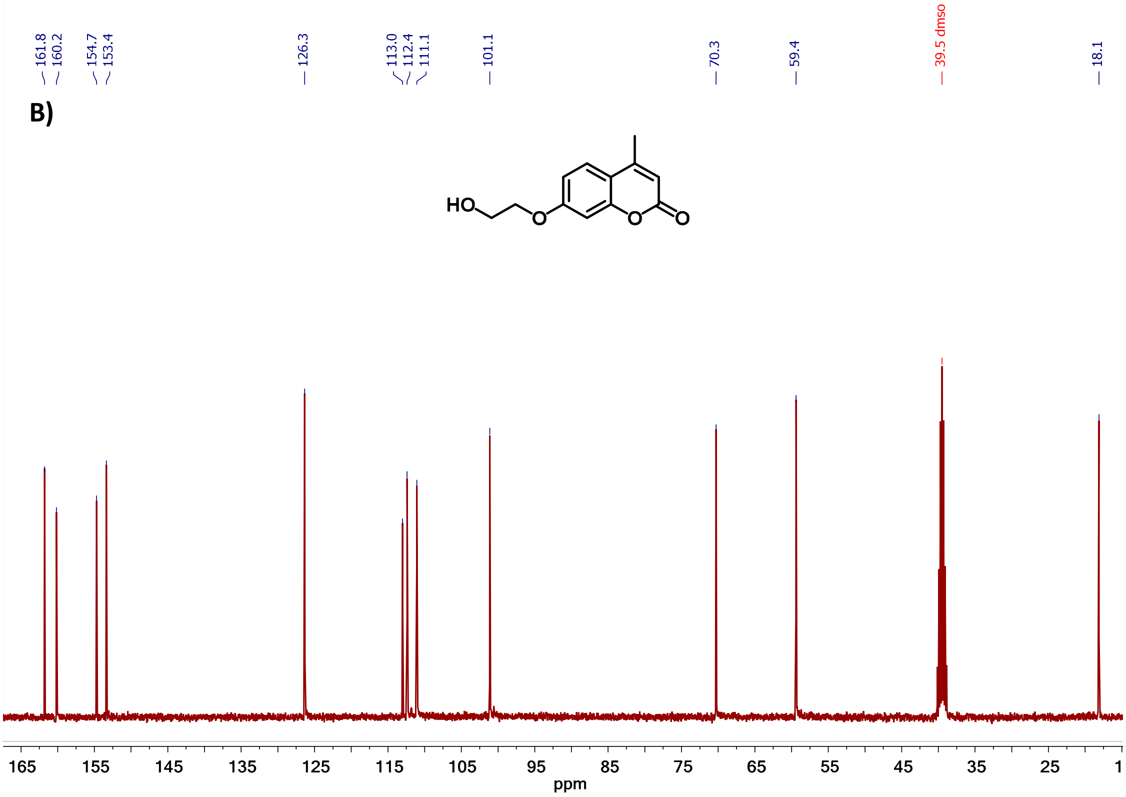

Figure S3: A) 1H-NMR and B) 13C-NMR spectra of HEOMC

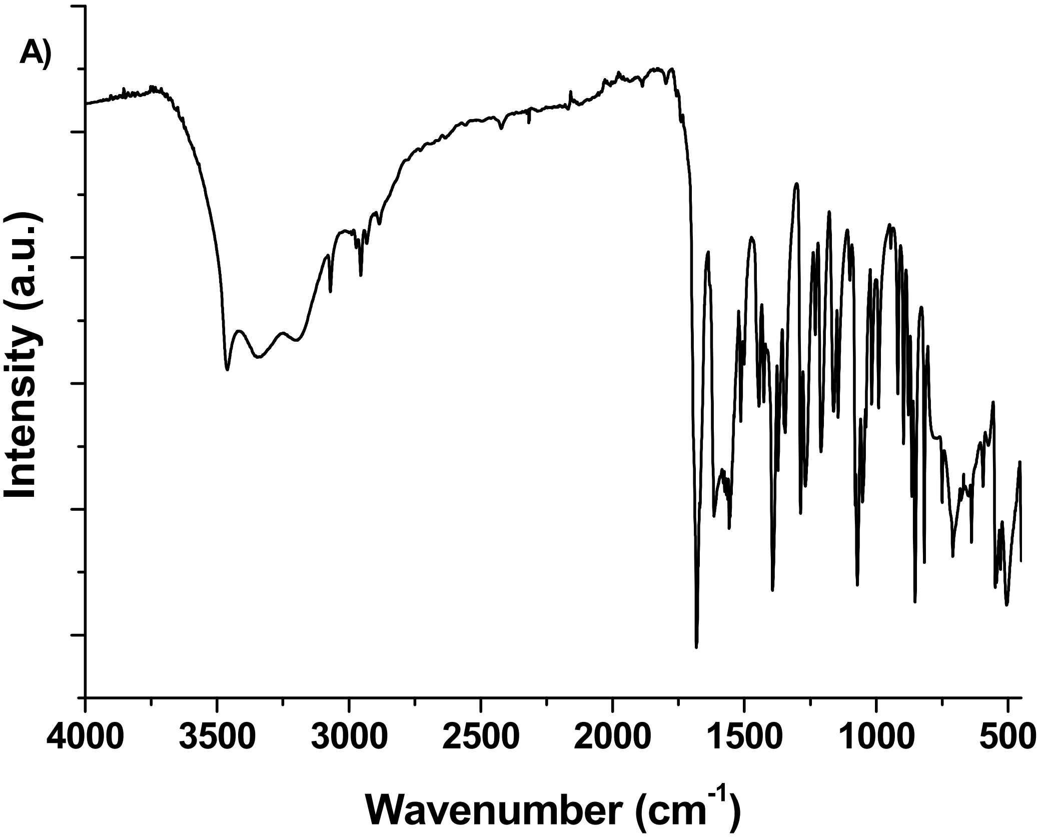

Compound Table

Compound Label	RT	Mass	Abund	Formula	Tgt Mass	Diff (ppm)
Cpd 1: C12 H12 O4	0.501	220.07388	60537	C12 H12 O4	220.07356	1.47

B)

Compound Label	RT	Algorithm	Mass
Cpd 1: C12 H12 O4	0.501	Find By Formula	220.07388

MS Zoomed Spectrum

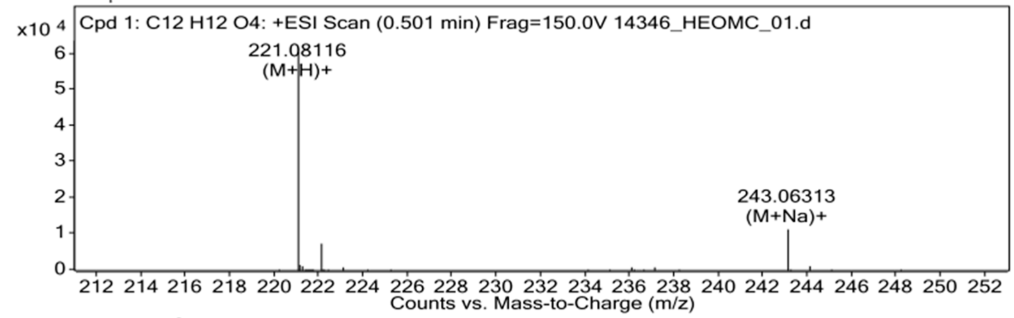

MS Spectrum Peak List

m/z	*Calc m/z*	Diff(ppm)	z	Abund	Formula	Ion
221.08116	221.08084	1.46		60537	C12 H13 O4	(M+H)+
221.13282				1786		
221.21828				1464		
221.26871				466		
222.08424	222.08423	0.05		7636	C12 H13 O4	(M+H)+
222.13774				379		
223.08575	223.08635	-2.68		899	C12 H13 O4	(M+H)+
225.19838				448		
243.06313	243.06278	1.42	1	11524	C12 H12 Na O4	(M+Na)+
244.06534	244.06618	-3.42	1	1412	C12 H12 Na O4	(M+Na)+

Figure S4: A) ATR-FTIR and B) Mass spectra of HEOMC

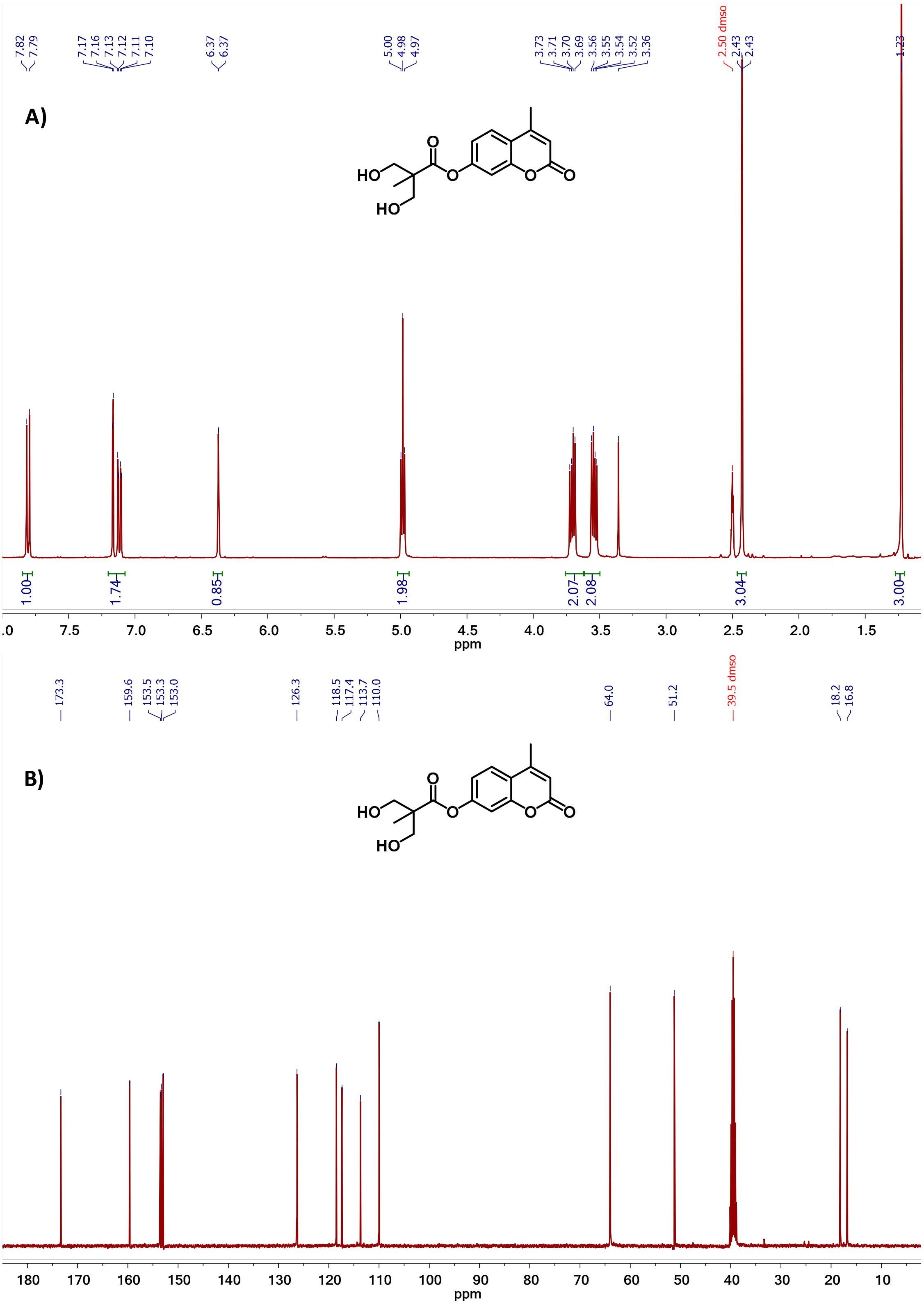

Figure S5: A) 1H-NMR and B) 13C-NMR spectra of DHMC

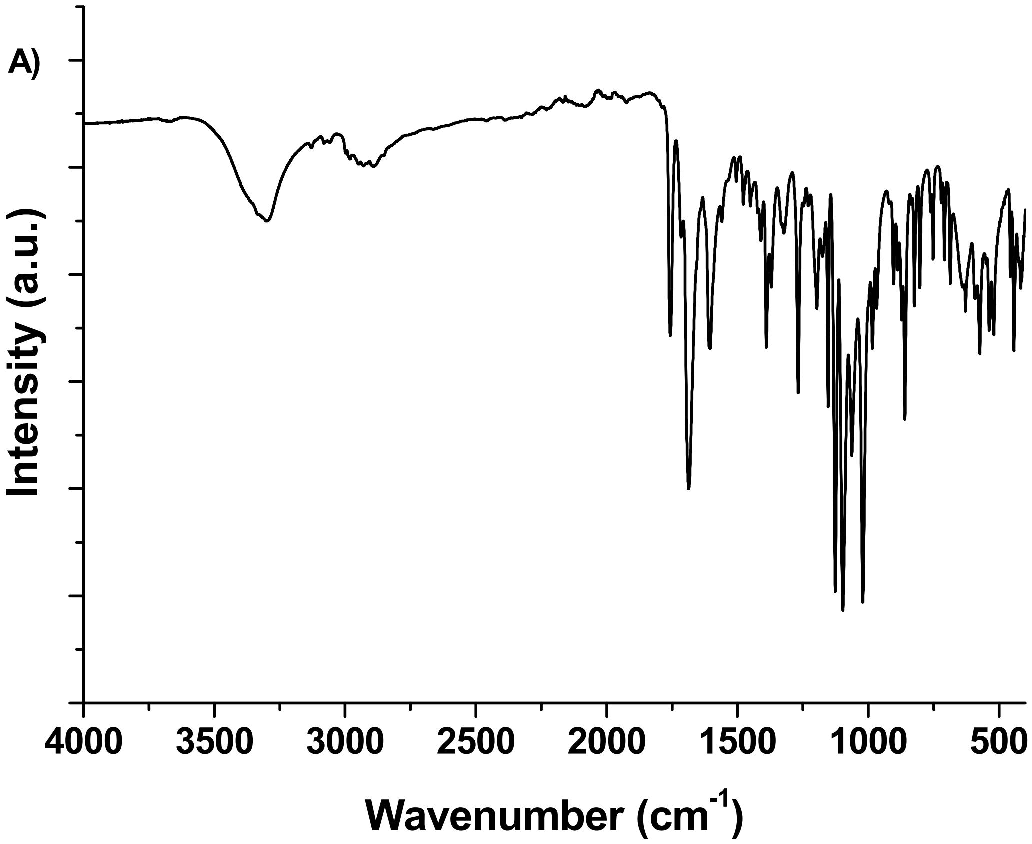

Compound Table

Compound Label	RT	Mass	Abund	Formula	Tgt Mass	Diff (ppm)
Cpd 1: C15 H16 O6	0.541	292.09355	40162	C15 H16 O6	292.09469	-3.9

B)

Compound Label	RT	Algorithm	Mass
Cpd 1: C15 H16 O6	0.541	Find By Formula	292.09355

MS Zoomed Spectrum

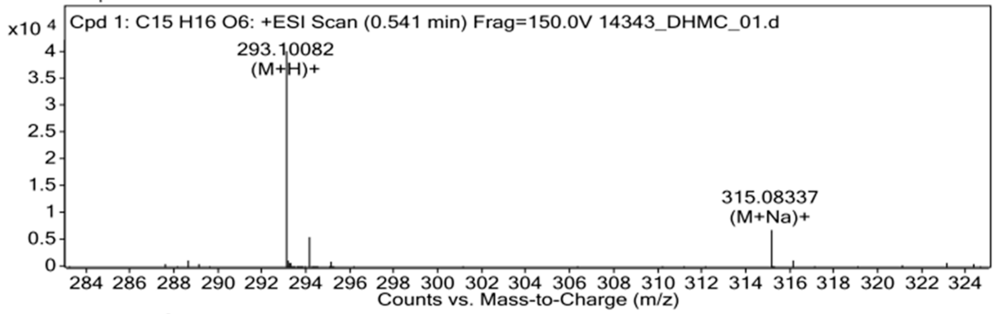

MS Spectrum Peak List

m/z	*Calc m/z*	Diff(ppm)	z	Abund	Formula	Ion
225.19548				74597		
225.24799				2017		
225.33407				1974		
226.19834				9363		
227.20273				962		
293.10082	293.10196	-3.9	1	40162	C15 H17 O6	(M+H)+
294.10405	294.10537	-4.46	1	5692	C15 H17 O6	(M+H)+
295.10475	295.10752	-9.4	1	1024	C15 H17 O6	(M+H)+
315.08337	315.08391	-1.71	1	6867	C15 H16 Na O6	(M+Na)+
316.08596	316.08731	-4.26	1	1284	C15 H16 Na O6	(M+Na)+

Figure S6: A) ATR-FTIR and B) Mass spectra of DHMC

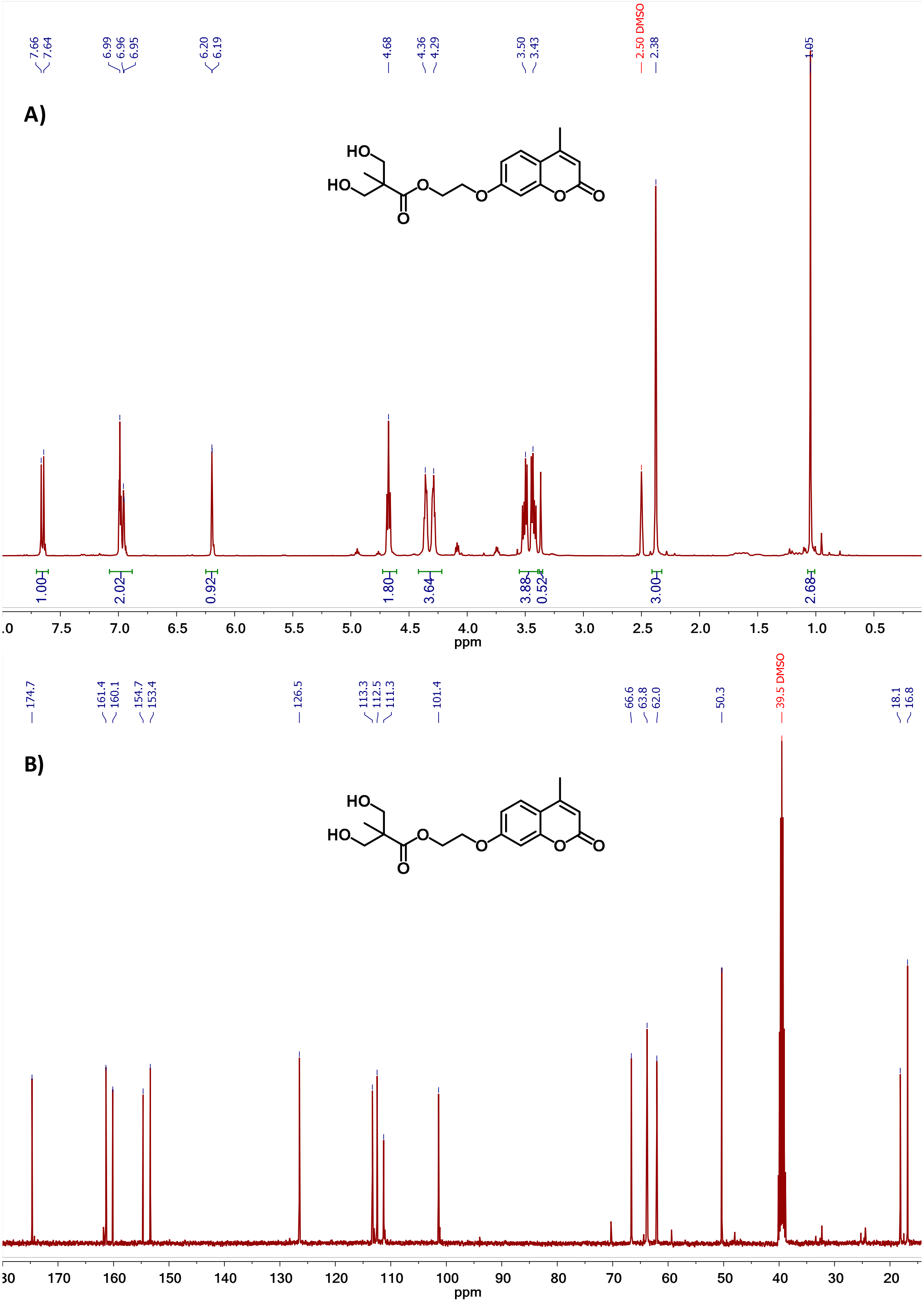

Figure S7: A) 1H-NMR and B) 13C-NMR spectra of DHEOMC

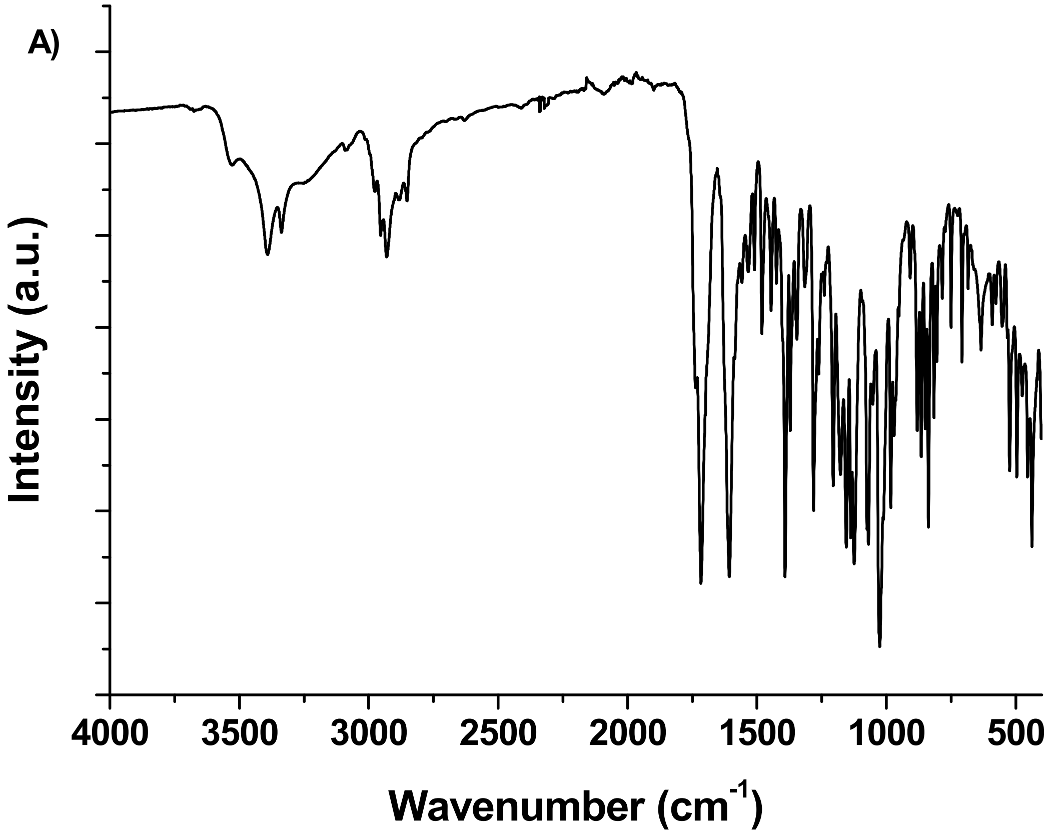

Compound Table

Compound Label	RT	Mass	Abund	Formula	Tgt Mass	Diff (ppm)
Cpd 1: C17 H20 O7	0.628	336.12093	47454	C17 H20 O7	336.1209	0.08

B)

Compound Label	RT	Algorithm	Mass
Cpd 1: C17 H20 O7	0.628	Find By Formula	336.12093

MS Zoomed Spectrum

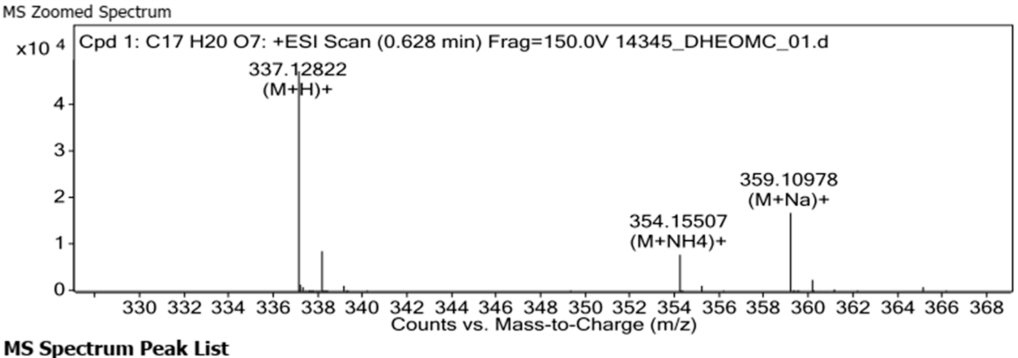

MS Spectrum Peak List

m/z	*Calc m/z*	Diff(ppm)	z	Abund	Formula	Ion
337.12822	337.12818	0.13		47454	C17 H21 O7	(M+H)+
337.18691				1584		
337.29695				1080		
338.13154	338.13158	-0.13		8643	C17 H21 O7	(M+H)+
339.13109	339.13381	-8.03		1391	C17 H21 O7	(M+H)+
354.15507	354.15473	0.96	1	8063	C17 H24 N O7	(M+NH4)+
355.15692	355.15802	-3.09	1	1265	C17 H24 N O7	(M+NH4)+
359.10978	359.11012	-0.95	1	17027	C17 H20 Na O7	(M+Na)+
360.11358	360.11353	0.16	1	2648	C17 H20 Na O7	(M+Na)+
361.11537	361.11575	-1.05	1	603	C17 H20 Na O7	(M+Na)+

Figure S8: A) ATR-FTIR and B) Mass spectra of DHEOMC

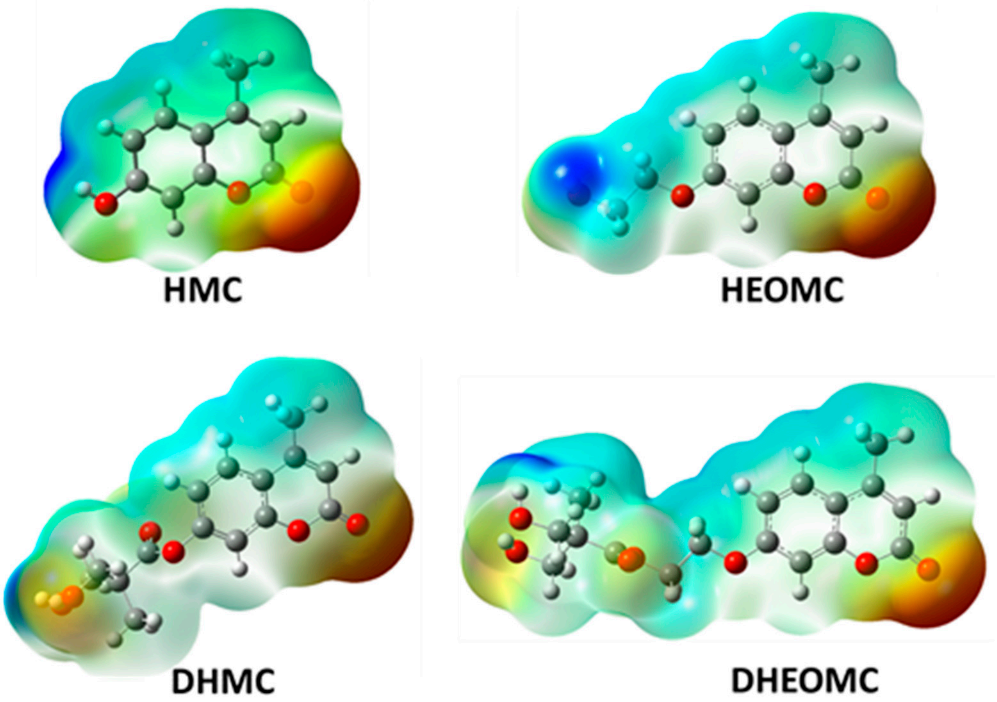

Figure S9: Electrostatic potential surfaces for the coumarins studied. Contour values range from -0.080 to 0.080 Hartree/e.

Table S1: Experimental and theoretical vertical excitation energies and oscillator strength for the first two excited states. The labels 1, 2, 1′ and 2′ correspond to HOMO, LUMO, HOMO-1 and LUMO+1, respectively.

Coumarin	Transition (weight)	Theoretical Excitation (nm)	Oscillator Strength	Experimental Absorption (nm)
HMC	S1: 1-> 1′ (0.95)	306	0.3980	317
	S2: 2-> 1′ (0.84)	278	0.0063	
	1 -> 2′ (0.14)			
HEOMC	S1: 1-> 1′ (0.96)	310	0.4660	319
	S2: 2-> 1′ (0.87)	282	0.0034	
	1 -> 2′ (0.11)			
DHMC	S1: 1-> 1′ (0.88)	295	0.3136	313
	2->1′ (0.07)			
	2->2′ (0.02)			
	S2: 2-> 1′ (0.81)	272	0.1408	
	1->1′ (0.07)			
	1->2′ (0.09)			
DHEOMC	S1: 1-> 1′ (0.96)	309	0.4934	318
	S2: 2-> 1′ (0.86)	281	0.0037	
	1 -> 2′ (0.11)			

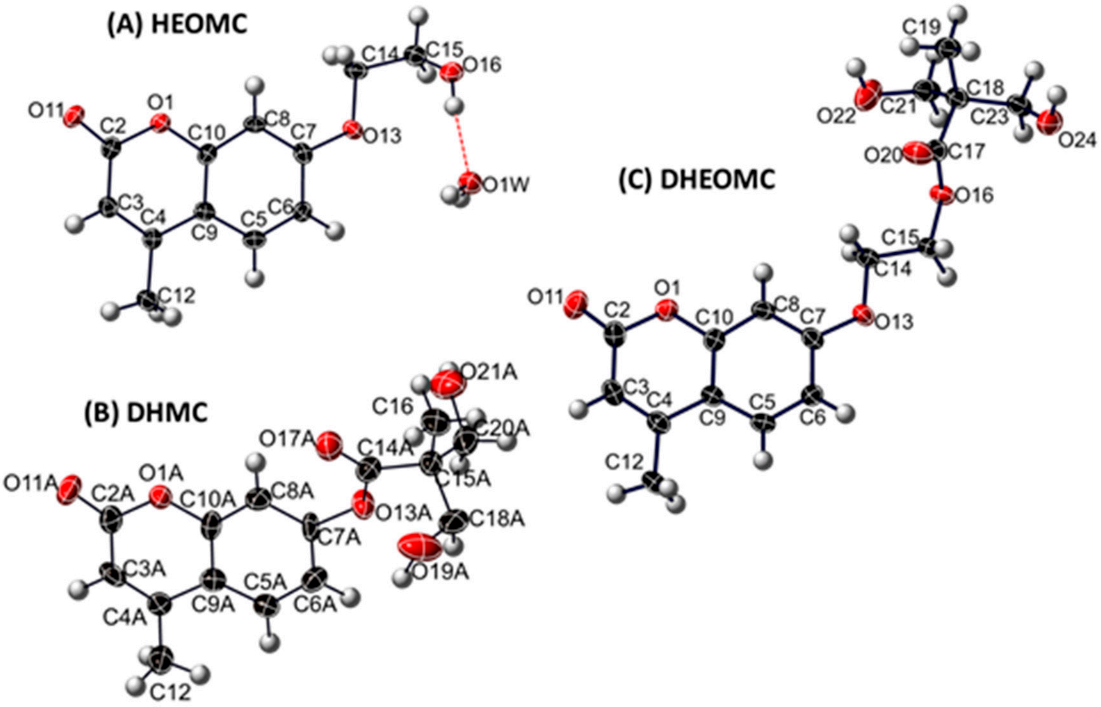

Figure S10: ORTEP view of the asymmetric units in (A) HEOMC, (B) DHMC and (C) DHEOMC depicted at the 50% probability level, together with atom labelling. Colour code: C, black, O, red, H, white.

Table S2: Geometrical parameters (Å, °) of intermolecular π-π interactions in HEOMC, DHMC and DHEOMC.

**Centroids (Cg)**	**Cg···Cg**	**ANG**	**Cg···plane**		**Slippage**
**HEOMC**					
**Cg1-Cg2**^{i}	3.574(2)	2.27(13)	3.441(2)	3.459(2)	0.900
**Cg2-Cg1**^{i}	3.574(2)	2.27(13)	3.459(2)	3.441(2)	0.966
**DHMC**					
**Cg1-Cg4**^{ii}	3.603(5)	3.9(4)	3.311(4)	3.349(4)	1.331
**Cg1-Cg4**^{iii}	3.571(5)	3.9(4)	3.525(4)	3.478(4)	0.806
**Cg2-Cg3**^{ii}	3.547(5)	3.7(4)	3.456(4)	3.496(4)	0.599
**Cg2-Cg3**^{iii}	3.602(5)	3.7(4)	3.385(4)	3.331(4)	1.371
**DHEOMC**					
**Cg1-Cg1**^{iv}	3.342(2)	0.03(10)	3.279(8)	3.279(8)	0.645

Cg = Centroid of the interacting ring. Cg1: O1, C2, C3, C4, C9, C10; Cg2: C5, C6, C7, C8, C10, C9.

Cg···Cg: distance between centroids; ANG: dihedral angle between planes containing both rings; Cg···plane: distance from one centroid to the plane containing the other ring. Slippage: distance between one centroid and its perpendicular projection to the plane containing the second ring. Symmetry codes: (i) -x, 1-y, 2-z; (ii) x, y, z; (iii) x, 1+y, z; (iv) 1-x, 1-y, 1-z.

Table S3: Geometrical parameters for O-H··O hydrogen bonds and C-H··O-type contacts in HEOMC, DHMC and DHEOMC.

D–H···A	D···A (Å)	Angle (°)
	**HEOMC**	
$O_{1W}–H_{1WA}···O_{11}^{i}$	2.874(3)	175
$O_{1W}–H_{1WB}···O_{16}^{ii}$	2.712(3)	175
$O_{16}–H_{16}···O_{1W}^{iii}$	2.716(3)	171
$C_{12}–H_{12C}···O_{11}^{iv}$	3.468(3)	167
$C_{8}–H_{8}···O_{11}^{v}$	3.573(4)	166
	**DHMC**	
$O_{19A}–H_{19A}···O_{41A}^{vi}$	2.673(14)	163
$O_{21A}–H_{21A}···O_{19A}^{vii}$	2.690(12)	158
$O_{49A}–H_{49A}···O_{51A}^{vii}$	2.694(12)	158
$C_{3A}–H_{3A}···O_{49A}^{viii}$	3.336(15)	152
$C_{5A}–H_{5A}···O_{17A}^{ix}$	3.446(15)	172
$C_{12}–H_{12}···O_{17A}^{ix}$	3.301(13)	158
$C_{20A}–H_{20B}···O_{11A}^{iv}$	3.397(14)	155
$C_{33A}–H_{33A}···O_{21A}^{viii}$	3.313(13)	150
$C_{35A}–H_{35A}···O_{47A}^{viii}$	3.470(16)	175
$C_{50A}–H_{50A}···O_{41A}^{x}$	3.387(15)	163
	**DHEOMC**	
$O_{22}–H_{22}···O_{20}^{xi}$	2.904(2)	137
$O_{24}–H_{24}···O_{22}^{iv}$	2.746(2)	163
$C_{3}–H_{3}···O_{11}^{xii}$	3.578(2)	171
$C_{21}–H_{21A}···O_{11}^{xiii}$	3.403(3)	132
$C_{19}–H_{19A}···O_{11}^{xiii}$	3.458(4)	137

D = donor; A = acceptor. Symmetry codes: (i) 1/2-x, -1/2+y, 3/2-z; (ii) x, y, -1+z; (iii) -1/2+x, 1/2-y, 1/2+z; (iv) -1+x, y, z; (v) 1-x, 1-y, 2-z; (vi) x, 1+y, z; (vi) x, -1+y, z; (viii) x, 1-y, -1/2+z; (ix) x, 2-y, -1/2+z; (x) 1+x, 1-y, ½+z; (xi) -1-x, -y, -z; (xii) 2-x, 1-y, 1-z; (xiii) –x, -y, -z.

Table S4: DSC results.

COUMARIN	DSC Heating				DSC Cooling			
	**Tm-onset (ºC)**	**Tm-peak (ºC)**	**Tm-endset (ºC)**	**ΔHm (J/g)**	**Tc-onset (ºC)**	**Tc-peak (ºC)**	**Tc-endset (ºC)**	**ΔHc (J/g)**
HMC	185.2	187.9	189.4	127.7	153.6	151.5	149.9	-93.2
DHMC	119.3	120.9	122.6	93.9	--	--	--	--
HEOMC	148.9	149.3	150.9	130.4	77.0	73.9	71.6	-71.3
DHEOMC	120.3	122.7	124.2	122.9	--	--	--	--

Tm-onset: onset melting temperature

Tm-peak: peak melting temperature

Tm-endset: endset melting temperature

ΔHm: melting enthalpy

Tc-onset: onset crystallization temperature

Tc-peak: peak crystallization temperature

Tc-endset: endset crystallization temperature

ΔHc: crystallization enthalpy

References:

1. Seoane Rivero, R.; Bilbao Solaguren, P.; Gondra Zubieta, K.; Peponi, L.; Marcos-Fernández, A. Synthesis, kinetics of photo-dimerization/photo-cleavage and physical properties of coumarin-containing branched polyurethanes based on polycaprolactones. *Express Polym. Lett.* **2016**, *10*, 84.

2. Jiang, X.; Wang, R.; Ren, Y.; Yin, J. Responsive polymer nanoparticles formed by poly (ether amine) containing coumarin units and a poly (ethylene oxide) short chain. *Langmuir* **2009**, *25*, 9629–9632.

3. Ling, J.; Rong, M.Z.; Zhang, M.Q. Coumarin imparts repeated photochemical remendability to polyurethane. *J. Mater. Chem.* **2011**, *21*, 18373–18380.

4. Yim, S.-H.; Huh, J.; Ahn, C.-H.; Park, T.G. Development of a novel synthetic method for aliphatic ester dendrimers. *Macromolecules* **2007**, *40*, 205–210.

5. Malkoch, M.; Malmström, E.; Hult, A. Rapid and efficient synthesis of aliphatic ester dendrons and dendrimers. *Macromolecules* **2002**, *35*, 8307–8314.
